# Rubabel: wrapping open Babel with Ruby

**DOI:** 10.1186/1758-2946-5-35

**Published:** 2013-07-24

**Authors:** Rob Smith, Ryan Williamson, Dan Ventura, John T Prince

**Affiliations:** 1 Department of Computer Science, Brigham Young University, Provo, Utah, USA; 2 Department of Chemistry, Brigham Young University, Provo, Utah, USA

**Keywords:** Chemoinformatics, Open Babel, Ruby

## Abstract

**Background:**

The number and diversity of wrappers for chemoinformatic toolkits suggests the diverse needs of the chemoinformatic community. While existing chemoinformatics libraries provide a broad range of utilities, many chemoinformaticians find compiled language libraries intimidating, time-consuming, arcane, and verbose. Although high-level language wrappers have been implemented, more can be done to leverage the intuitiveness of object-orientation, the paradigms of high-level languages, and the extensibility of languages such as Ruby. We introduce Rubabel, an intuitive, object-oriented suite of functionality that substantially increases the accessibily of the tools in the Open Babel chemoinformatics library.

**Results:**

Rubabel requires fewer lines of code than any other actively developed wrapper, providing better object organization and navigation, and more intuitive object behavior than extant solutions. Moreover, Rubabel provides a convenient interface to the many extensions currently available in Ruby, greatly streamlining otherwise onerous tasks such as creating web applications that serve up Rubabel functionality.

**Conclusions:**

Rubabel is powerful, intuitive, concise, freely available, cross-platform, and easy to install. We expect it to be a platform of choice for new users, Ruby users, and some users of current solutions.

## Background

Despite the fact that chemoinformatics tools have been developed since the late 1990s [1], the field has yet to rally in support of a single library. The intricacies of the libraries combined with the low-level programming prowess required for these languages present a considerable barrier to adoption by less programming-oriented practitioners. What’s more, the competing libraries don’t share complete coverage of implemented tasks, meaning that the practitioner, who may be struggling with the language barrier, has to shoulder the additional burden of being well versed in the differences between the libraries, including different APIs, different IO interfaces and different data type standards.

The Cinfony project [2] is an attempt to offer high level access to three major existing chemoinformatics libraries from Python [3], a high-level scripting language [4]. Cinfony’s use of Python greatly reduces the number of lines of code required for a broad range of chemoinformatics tasks. Though it allows the user to access the functionality of the component libraries from one Python script, Cinfony does not automatically manage underlying data types nor the choice of which library to use for which function. This allows users more control over how Cinfony works. However, as the authors acknowledge, it requires users to have an intimate knowledge of the component libraries in order to manage what data types, conventions, and operations can be performed by each of the three libraries it wraps. Despite the success of Cinfony, there is still a need for simplified, high level access to common chemoinformatics tasks.

Since most common tasks are available in any single chemoinformatics library, wrappers for single tool kits are widely used. Because these wrappers interface into a single library, they have the potential for simpler interfaces and easier extension.

Pybel [5,6], a Python toolkit inspired by the Daylight project [7,8], wraps the chemoinformatics library Open Babel [9]. Pybel has an active user base and is an actively developed high-level solution to the accessibility problem of the available chemoinformatics libraries. Still, Pybel’s implementation in Python may not be the most intuitive interface for new users, who may not be strong programmers, or for Rubyists, who will miss multi-lined lambdas, simple extension (i.e, open-classes), and Rubygems, Ruby’s streamlined add-on installation tool [10].

In addition to Pybel, other attempts have been made to make open source chemoinformatics libraries more accessible. Indigo Python, a Python wrapper bundled with the Indigo open source chemoinformatics library [11], is a substantial improvement over the C++ library it wraps in terms of reduction of lines of code (LOC) needed to implement common tasks. RDKit [12] is a C++ library that has a Python wrapper and provides substantial reduction of LOC over direct access to the underlying C++ library. Most other available toolkits are either proprietary (such as OpenEye [13] and CACTVS [14]) or have not yet been documented and developed to maturity.

Ruby has penetrated the applied sciences where the need for a concise but powerful language meets appreciation for an easy learning curve [15,16]. A minimal learning curve, concise coding, and powerful language paradigm have made Ruby an attractive option for coding bioinformatics tools, such as BioRuby [17].

For those who are not comfortable enough with programming to use the current tools, for those who prefer the Ruby way, and for those who want to do more with fewer lines of code, we present Rubabel. Rubabel offers a convenient, intuitive molecule-centric interface and facile intra-molecular navigation with minimal lines of code per task. It is an easily installed, actively developed project with a substantial amount of implemented functionality and an arbitrarily accessible extension mechanism for customization.

## Implementation

**Figure 1:**
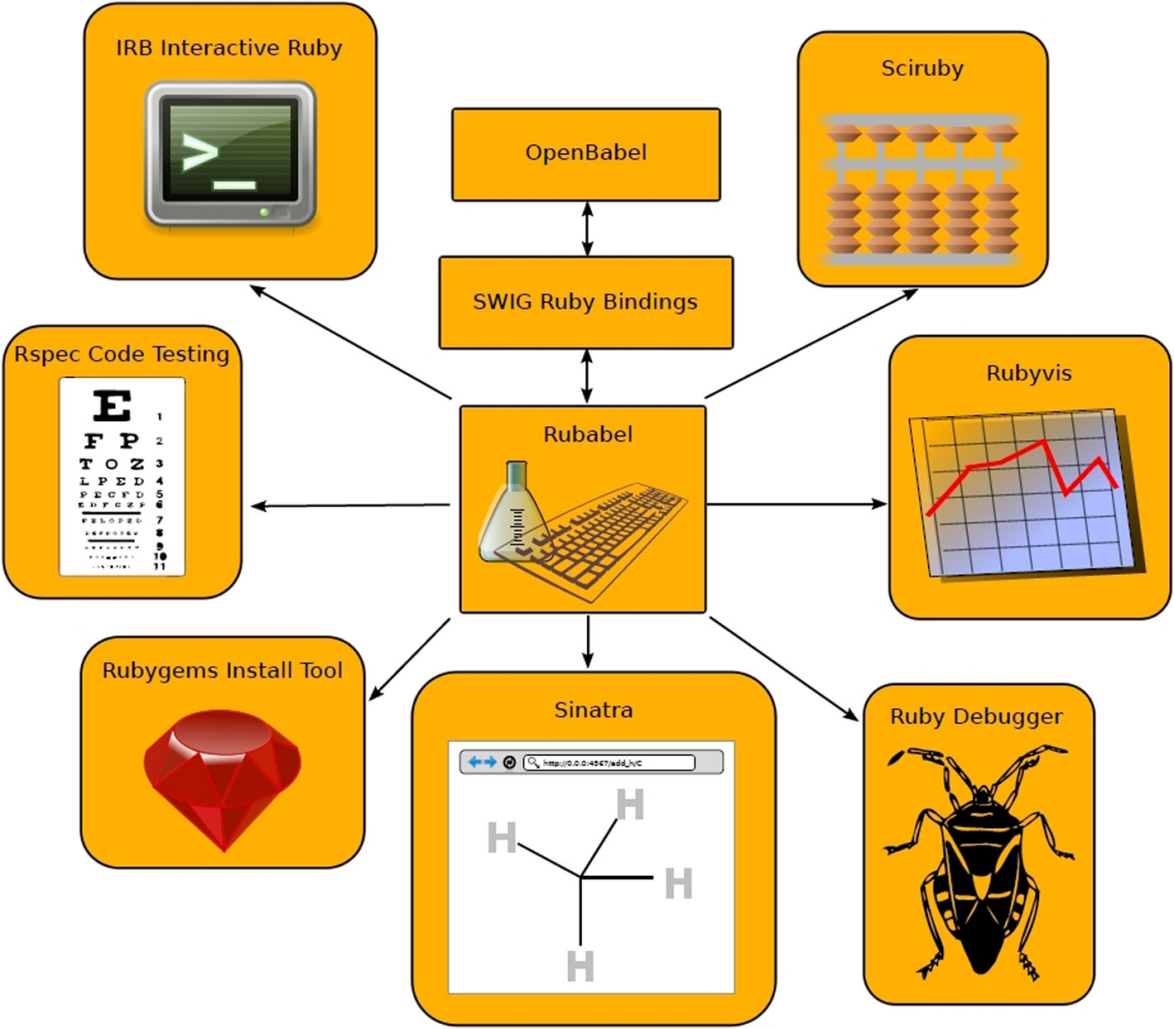
**Rubabel Architecture.** Rubabel reorganizes Open Babel functionality in an object-oriented architecture via the Ruby SWIG bindings and adds significant novel functionality. Additionally, Rubabel facilitates the integration of Ruby’s substantial library of extensions. These include debugging tools (Ruby Debugger), code testing (Rspec), graphic visualizations (Rubyvis), rapid dissemination of tools (Rubygems), web interfaces (Sinatra), and scientific libraries (Sciruby).

Rubabel’s architecture interfaces with Open Babel through its Ruby SWIG bindings (see Figure 1). Open Babel is an established chemoinformatics library written in C++ that provides a wide array of chemoinformatics functionality for programmatic or command line usage. Open Babel supports 111 chemical file formats, including SMILES, SMARTS [18], and InChI. It has fingerprint support, bond perception, atom typing, image representation capabilities, stereochemistry recognition, and forcefields management, among other features. Its wide use is evidenced by its adoption by over 65 software applications, libraries, web applications, and databases [11].

Open Babel’s acceptance rests at least partly on its SWIG bindings [19] which allow it to be accessed from languages other than C++. The bindings provide handles for accessing the internals of Open Babel.

### Ruby SWIG bindings

For those who are less confident in C++ programming or aren’t familiar enough with the code base to know the command line composition for their desired task, Open Babel’s Ruby SWIG bindings provide an alternative solution. Although the bindings technically allow access to Open Babel from Ruby, it quickly becomes evident that the user is not convincingly spared from C++. An intimate understanding of Open Babel’s implementation architecture is required for many if not most tasks, and in some cases an almost line-for-line translation from C++ to Ruby is necessary. For example, Listing Ruby SWIG bindings shows how to instantiate a molecule from a SMILES string with the Ruby bindings.

#### Listing 1 Creating a molecule from a SMILES string with Open Babel Ruby bindings

Rubyists will notice that this code seems strikingly more like C++ than Ruby. Moreover, note that despite the uncharacteristic simplicity of this example, the user still needs to understand explicit details of the Open Babel architecture including the OBMol and OBConversion objects and modification methods for OBConversion. For more complex but typical examples, such as highlighting a substructure within a molecule in an image, the LOC required are comparable to C++. Adding Ruby-style objects and idioms to the SWIG bindings is the obvious next step toward improving upon the Ruby SWIG.

Rubabel is much more than a wrapper that ports Open Babel functionality to Ruby. Rubabel organizes the Open Babel objects into a more intuitive structure and extends the available behavior in a manner consistent with Ruby idioms, which is beneficial to experienced Rubyists and non-programmers alike, who both will find the interface intuitive and straightforward.

### Rubabel: augmentations to open babel

**Figure 2:**
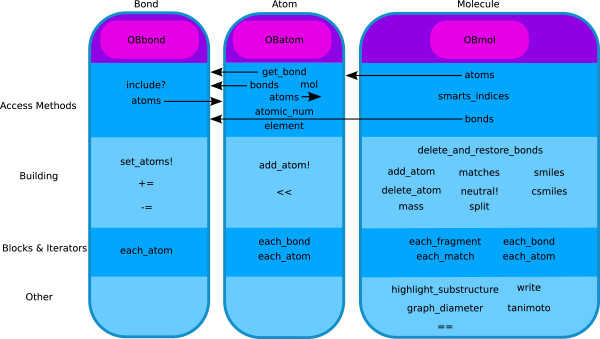
**Novel functionality in Rubabel.** Besides providing access to native Open Babel functions, Rubabel provides a host of novel functionality.

Rubabel’s objects are designed to be intuitive. Table 1 lists the Rubabel objects which wrap Open Babel functionality. Although the names for these objects correspond to similarly named objects in Open Babel, Rubabel augments Open Babel functionality substantially. Figure 2 lists some of the novel methods offered by Rubabel, some of which are not available in any other chemoinformatics toolkit. Additionally, every Rubabel object has full access to the behavior provided by the underlying Open Babel object.

**Table 1:** Rubabel objects


Molecule	Wraps Open Babel’s OBmol object. Adds the ability to intelligently manipulate molecules as strings, transfer to and from lists of atoms and bonds, add and modify atoms, explicit and general molecular matching, iterate over bonds or atoms, copy molecules, png representation of the molecule, and fingerprinting.
Atom	Wraps Open Babel’s OBatom object. Adds accessibility conveniences such as the ability to seamlessly create or access an atom as an atomic number, the ability to intrinsically iterate through bonds and pass blocks to iterating loops, and the ability to iterate through and optionally execute a block of code for each atom bonded to the current one.
Bond	Wraps Open Babel’s OBBond object. Adds an accessor for a list of attached atoms, a seamless enumerator for attached atoms, the ability to execute a block of code for each attached atom, and the ability to easily check if a given atom is connected with this bond.
Smarts	Wraps Open Babel’s smarts pattern object.

Rubabel’s object organization is an intuitive restructuring of Open Babel’s architecture. For example, molecule printing logic found in an external object in Open Babel is moved inside the Molecule object. Rubabel’s objects have extended novel capabilities (detailed below).

### Rubabel: Ruby idioms for concise and convenient code

Ruby is a language designed to be easy to use, intuitive, and fun. We designed Rubabel to embody as many of these admittedly subjective qualities as possible by designing Rubabel object behavior in ways consistent with established Ruby idioms for object behavior.

#### Object orientation

Rubabel’s object-oriented paradigm defines behaviors for the objects with which they interact. For example, Open Babel’s Tanimoto coefficient logic will always apply to molecules, so in Rubabel that functionality is built into a method on the Molecule object. Similarly, in Open Babel, write methods for drawing molecules in image files are located in the code base as discrete functions. However, object-oriented methodology dictates that the objects themselves—not external modules—should define how they are printed. In Rubabel, write methods are defined for Molecule. Another example of linking behavior to the objects modified can be found in Rubabel iterators. In Open Babel, each object has its own iterator type as a separate object. In Ruby, iterators are implicit and connected to the object iterated over. There is no need to look up behavior because Rubabel’s iterators work exactly like iterators over native Ruby objects.

By following the object-oriented paradigm, users can instantly know what behavior is defined on any object by simply typing <object name>.methods in an interactive Ruby console. Non-object-oriented code requires digging through documentation or, if there isn’t any, sourcecode. Both options are unappealing due to the time commitment, while the latter option is inaccessible to non-programmers.

Listing Object orientation is illustrative of how object-orientation facilitates more intuitive code. Through Rubabel’s explicit Bond object, one can access a bond (line 3), upgrade its order (line 3), and downgrade its order (line 4). One would expect that the plus and minus operators to define the syntax to increase or decrease a bond’s order. With Rubabel, they are.

##### Listing 2 Ad-hoc bond modification

Object-orientation reduces lines of code. When considering an SD file, it seems reasonable to think about each entry in the file as a Molecule object. With Rubabel, you can do exactly that by iterating through each Molecule object in a file. Listing Object orientation shows how to open an SD file and print out each molecule whose weight is in the range (300,400) in just one line of code.

##### Listing 3 Report how many SD file records are within a certain molecular weight range

#### String idiom

To assist in convenience and minimize syntax lookup time, the Ruby idiom for strings is engineered for frequent exposure in Rubabel. For example, the Molecule object can be implicitly treated as a string, allowing splitting and matching operations that are concise and intuitive. For example, the user can collect all atoms with two bonds (excluding implicit hydrogen atoms) and append a carbon to the first atom in the filtered collection (see Listing String idiom).

##### Listing 4 Matching in Rubabel

Additionally, Rubabel implements both split and append methods for the Bond object that mirror the same behavior defined in Ruby strings. Listing String idiom shows an example of splitting bonds. Lines 2-4 create a molecule, find each single bond that links a carbon atom to an oxygen atom, then splits those bonds. Line 5 appends a carbon and an oxygen atom to mol using atomic numbers with the append function. Line 6 does the same using the element name.

##### Listing 5 Splitting and appending Molecule objects

Because molecules are treated as lists of atoms, you can quickly and easily access and modify specific atoms in a molecule. Listing String idiom demonstrates adding an ethyl group atom-by-atom to the first carbon atom by indexing into the Molecule (line 3).

##### Listing 6 Constructing a molecule atom-by-atom with the Ruby string idiom in Rubabel

No other toolkits have equivalent functions to the string idiom in Rubabel.

#### Access methods

Rubabel is designed to simplify common IO tasks to provide the shortest path to chemoinformatics functionality. Rubabel allows creation of Molecule objects from every format Open Babel accepts, including SMILES strings (see Listing Access methods). Note that Rubabel requires only one line where the SWIG code requires 4 (compare with Listing Ruby SWIG bindings).

##### 7 Creating a molecule from a SMILES string with Rubabel

Efficiency in accessing objects is very important to reducing LOC and increasing intuition. Listing Access methods gives some examples of object traversal in Rubabel, highlighting the amount of processing that can be done with very few lines of code in Rubabel. With very few lines of code and intuitive method names (select, find, reject), the user is able to conduct significant operations on newly created molecules. Lines 2-3 create a molecule then find the atom(s) that contain a double bond. Line 4 finds all the single- and double-bonded oxygen atoms in the molecule. Line 5 first finds all oxygen atoms, then removes from that list those that are bound to a carbon atom, yielding the peroxy oxygen.

##### Listing 8 of objects in Rubabel

#### Building

Rubabel offers multiple novel methods that assist in building and modifying molecules and bonds. Several, including the bond order increment/decrement operator, split, and match functions were already highlighted. Additionally, the Molecule object defines adding and removing atoms, as well as a mass method that calculates the mass of the molecule taking into account the charge state.

#### Blocks

Blocks are dynamic sections of code with open scope, sometimes several lines long, that allow injection of specific behavior into otherwise generic methods. This allows greater code reuse, concise code, and places custom logic next to the object it modifies instead of in a separate function or an external library. Consider Listing Blocks. By using find parameters in a block, Rubabel obtains a specific molecule in an SDF file in a more concise manner than RDKit, a Python chemoinformatics toolkit, which requires more control structure and logic (see Listing Blocks).

##### Listing 9 Finding a certain molecule in an SDF file in Rubabel

##### Listing 10 Finding a certain molecule in an SDF file in RDKit/Python

Additionally, blocks make for easier synonymous code—code that is different syntactically but equivalent functionally. This increases the likelihood that a non-expert user can ascertain the syntax of desired operations with minimal reference to documentation while allowing more experienced users the freedom to use coding styles they are familiar and comfortable with.

##### Listing 11 Rubabel provides synonymous syntax

In addition to the two examples given here, Listings Object orientation and Access methods use blocks as well (lines 2 and 3-5, respectively). They are powerful tools not available in languages like C++ and Python.

#### Custom behavior

We have provided explicit Molecule methods for common tasks such as Tanimoto coefficient calculation, substructure highlighting, and graph diameter measurement. In the likely event that users need custom extended behavior in Rubabel, they can take advantage of what are known as Ruby open classes. Objects in Ruby are more accessible to behavior modification than in some other languages. Writing custom behavior into Rubabel is analogous to using a plugin. Although Open Babel has a plugin mechanism which allows external code to be integrated into the toolkit, usage is not trivial. In contrast, Rubabel can be modified and accessed with ease using Ruby’s open classes. A class is open when it allows any external code to add or modify functionality in the local scope. For example, the authors are currently developing a plugin for Rubabel that defines fragmentation behavior for lipid molecules. They require the molecule behavior defined by Rubabel and also need to add descriptions of how lipids fragment in order to accomplish their task. With Rubabel, this is as simple as adding a few lines in a new Ruby file, as in Listing Custom behavior.

##### Listing 12 Defining custom behavior for Rubabel: it is arbitrarily simple to add custom behavior to Rubabel by leveraging Ruby’s open classes

By using Rubabel, custom behavior can be defined and shared amongst lab groups and colleagues rapidly and easily.

### Rubabel: extensions from Ruby

Rubabel has access to the Ruby community’s many actively developed extensions (see Figure 1 and Table 2 for examples). These extensions and the many more like them provide diverse and useful benefits such as quicker programming, easy debugging, and easy installation. Some Ruby add-ons, like Sinatra [20], a concise web application framework, and Rspec [21], a test-driven development suite, have no equivalent of which we are aware in other languages such as Python.

**Table 2:** Ruby extensions accessible to Rubabel

**Extension**	**Possible application with Rubabel**
Sinatra [20], a web application framework	Quick and easy webapp GUI for Open Babel, allowing multi-platform point and click chemoinformatics
Sciruby [16], a scientific library	Plotting, statistical tools, access to R programming language for Rubabel results
Rubyvis graphical library [22]	Open ended graphical software to make clean representations of numerical data
IRB, the interactive Ruby shell	Quick access to Rubabel and Open Babel from a terminal
Rspec, an automated code testing library [21]	Automated unit tests for software built with Rubabel (No Python equivalent due to Ruby’s block ability)
Ruby debugger [23]	Step into executed code with a live IRB session to ferret out bugs
Rubygems, a distribution tool [10]	Easily distribute and integrate applications written with Rubabel with a one-line install

Ruby has an active community of contributors who are constantly developing open source tools and frameworks.

#### Building a Rubabel web app in Sinatra

As an example of the capabilities of these extensions, consider Sinatra. Using Sinatra, it is possible to give practitioners online access to Rubabel in very few lines of code. Applications can easily be developed to serve up both the native functionality of Rubabel as well as custom functionality developed as needed. To demonstrate the brevity of code required, consider the task of adding explicit hydrogen atoms to a molecule and printing the SVG image of the new molecule. Assuming that the user has a standard install of Ruby, which includes Rubygems and the prerequisites for Open Babel, the entire environment for Sinatra and Rubabel can be installed in one line (see Listing Building a Rubabel web app in Sinatra).

##### Listing 13 Installing Rubabel and Sinatra

The functionality for the web app requires only five lines of code (see Listing Building a Rubabel web app in Sinatra). We place these in the file mol_h.rb.

##### Listing 14 A web application that adds a hydrogen atom to a molecule

**Figure 3:**
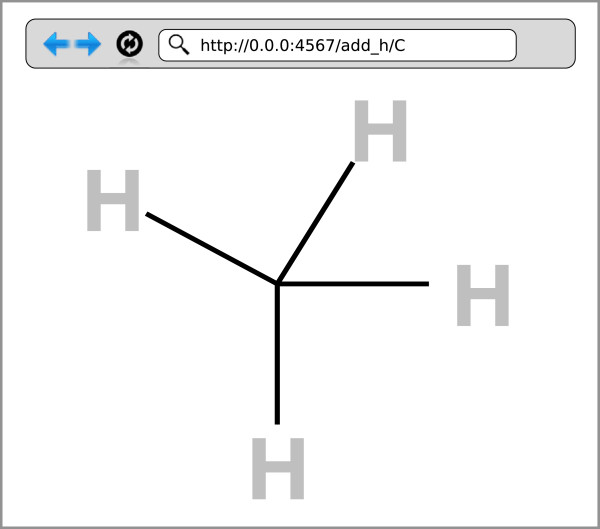
**Custom web apps with Rubabel.** The Sinatra toolkit for Ruby allows easy web access for Rubabel and add-ons.

Now, to invoke our web server locally, we simply open a terminal and write: ruby mol_h.rb The web service is now available. Now we can convert a smiles string to a molecule, then add an explicit hydrogen and print the resulting molecule simply by typing http://0.0.0.0:4567/add_h/C into a browser window. This results in a web page that displays the SVG of the resulting molecule (see Figure 3). The address http://0.0.0.0:4567/ accesses the local web server. The argument add_h tells Rubabel that we want to add a hydrogen onto the last argument of the url, the SMILES string C.

The simplicity of this example readily extends to virtually all facets of Rubabel.

#### The interactive Ruby shell (IRB)

Though space will not permit an exhaustive consideration of all Ruby extensions that can be used in conjunction with Rubabel, the interactive Ruby shell (IRB) is of special import. As with languages like Python, Ruby’s interactive shell allows users a ready sandbox to run quick experiments, test syntax, or debug their scripts. IRB can be installed (provided the user has Rubygems) by typing gem install irb. Simply enter the IRB environment (irb at the terminal) and type require ‘rubabel’ and all of Rubabel’s functionality is accessible in an interactive terminal. This is particularly useful given the number of tasks that Rubabel can accomplish in just one line. Rubabel in IRB provides an interactive sandbox to experiment in realtime with instant feedback—a refreshing alternative to stringing together guess-and-check command line arguments. As mentioned before, this is also a fantastic and fast way to look up (via <object name>.methods) or check syntax.

## Results and discussion

To provide a quantitative analysis of Rubabel compared to existing tools, we compare the required number of lines of code (LOC) from the Chemistry Toolkit Rosetta Wiki tasks [24]. The CTRwiki provides code snippets for 18 common chemoinformatics tasks for more than 17 toolkits in various programming languages. Since there are several toolkits with only one or two solutions we consider only open source solutions with at least 5 of the CTR tasks documented. We use the CTR tasks implemented as a quantitative measure of accessibility to functionality and not necessarily as an absolute measure of what is or is not implemented in a toolkit. There are many toolkits out there, with varying off-the-shelf capabilities and documentation. Since these toolkits are wrappers with access to the underlying libraries, it is possible, given enough time and code, to do anything in them that the underlying library does. However, the point of this comparison is to show off-the-shelf accessibility, scope, and verbosity. From a user perspective, having a listing of a core set of basic behaviors is useful not only from a comparative standpoint, but because of the appeal of off-the-shelf code to accomplish a standard task. That the CTR tasks are acceptable and demonstrative is suggested by the number of platforms that have adopted them, including Open Babel, which has provided a tutorial showing how to do each of them. Though a lack of a CTR entry for a given method does not indicate that the task is not possible, it means that the recipe for the task is not available in this convenient location and is thus harder to obtain than the tasks that are posted there. The CTR tasks also provide a convenient and established set of functionality to demonstrate a baseline set of features provided in Rubabel.

**Figure 4:**
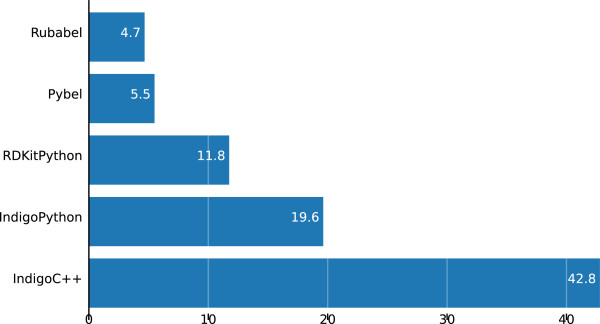
**Average lines of code per CTR task.** On average, Rubabel requires fewer lines of code than any other toolkit.

**Figure 5:**
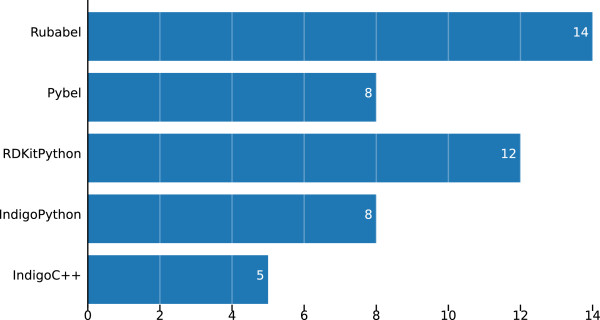
**Number of CTR tasks listed.** Rubabel has more tasks listed than any other toolkit.

**Figure 6:**
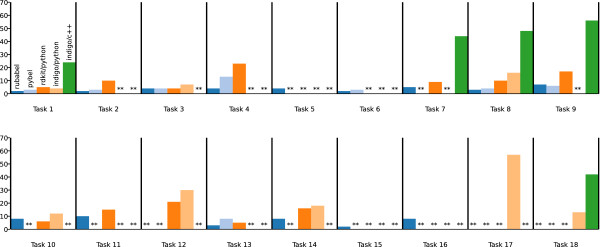
**Lines of code per CTR task.** Rubabel has more CTR tasks listed than any other toolkit, and also requires fewer lines of code than any other toolkit on every task except task 9, where Pybel uses one fewer line of code, and task 10, where rdkit/Python is slightly more concise.

Rubabel dominates Indigo C++ in number of lines of code per task, and is more concise than other scripting language toolkits (see Figure 4). Rubabel has fewer lines of code per task on average than Pybel. Rubabel also implements almost double the number of CTR tasks as Pybel (see Figure 5), and when broken out by task, we can see that Rubabel is more concise than Pybel on each task for which they are both implemented save one (task 9) (see Figure 6). Moreover, Rubabel is more concise than all other methods for each task save rdkit/Python on task 10.

Rubabel has some features which users may find useful that are not available in Pybel. These include an explicit Bond object and the associated functionality, simpler atom interrogation, enumerable atoms and bonds, intuitively named wrapped output and input options (obviating the need to dig through Open Babel documentation to enumerate them), more Molecule object modifications (e.g. adding hydrogens at a specific pH), and simpler output (Rubabel infers the output format from the filename). Additionally, there are several extensions written in Ruby that do not yet have equivalents in Python (see Table 2).

Rubabel is open source software released under the liberal MIT license. The license and source code, as well as instructions on how to install, are found at https://github.com/princelab/rubabel. The project is available as a Ruby gem [10], which makes it easy to install. For those who already have Ruby, Rubygems, and Open Babel’s prerequisites installed, Rubabel and all requirements (including Open Babel) can be installed with one line: gem install rubabel. Rubabel can also be downloaded and built from source. The instructions for this are available on the github site mentioned above.

## Conclusions

Chemists are not necessarily computer scientists. The more concise, clear, and accessible a toolkit is, the less time they spend learning syntax and the more time they spend solving chemistry problems. Ruby is designed to be intuitive, concise, and powerful. Rubabel wraps Open Babel in a way that is true to these qualities. Rubabel provides more intuitive object organization than Open Babel and provides extra functionality designed to streamline code writing by limiting both the time necessary to look up function syntax and the number of lines of code required. Rubabel also provides access to the many open source extensions available for Ruby. Rubabel’s concise and intuitive design makes common chemoinformatics tasks readily accessible from scripts, interactive shells, or custom applications in few lines of code and with less time spent learning APIs. Intentionally intuitive design, concise code idioms, and simplified common tasks make Rubabel appealing to Rubyists, non-programmers, and a segment of the users of other platforms.

## Availability and requirements

**Project name:** Rubabel

**Project home page:**https://github.com/princelab/rubabel

**Operating System(s):** Platform independent

**Programming language:** Ruby

**Other requirements:** Open Babel’s Install Requirements, Rubygems

**License:** MIT

**Any restrictions to use by non-academics:** None

## Competing interests

The authors declare that they have no competing interest.

## Authors’ contributions

JP is the founding developer of Rubabel. RS and RW extended Rubabel. DV provided valuable guidance and editing. All authors read and approved the final manuscript.
